# Editorial: Lipid Metabolism and Transport in CNS Health and Disease

**DOI:** 10.3389/fphys.2021.768417

**Published:** 2021-10-13

**Authors:** Kimberley D. Bruce, Hussein N. Yassine, Alfred N. Fonteh

**Affiliations:** ^1^Division of Endocrinology, Metabolism, and Diabetes, University of Colorado Anschutz Medical Campus, Aurora, CO, United States; ^2^University of Southern California, Los Angeles, CA, United States; ^3^Huntington Medical Research Institutes, Pasadena, CA, United States

**Keywords:** lipids, lipoproteins, HDL, microglia, neurodegeneration, PUFAs

The brain is mostly composed of lipids, yet lipid metabolism and processing in the brain are incompletely understood. With the advancement of higher-resolution technologies, we are becoming increasingly aware that even slight changes in lipid and lipoprotein processing in the brain can precede or even predict disease. This Research Topic gathers contributions that highlight how lipids are transported; across the blood-brain barrier (BBB), by microglia, stored as lipid droplets (LDs), or transported in the form of APOE-containing high-density lipoprotein particles, and the importance of lipid composition in predicting disease. The compiled group of articles provides a better understanding of these processes, which may lead to the development of more targeted strategies to diagnose and treat neurodegenerative disorders (NDs).

In the first article, Huguenard et al., describe the long-standing need for biomarkers that can diagnose mild Traumatic Brain Injury (mTBI) and Post-Traumatic Stress Disorder (PTSD). Building on previous work detecting chronically altered brain-associated phospholipids in the blood of subjects with mTBI, the authors reason that the analysis of peripheral lipids may also identify patients with mTBI and PTSD. Using liquid chromatography-mass spectrometry (LC-MS) to examine lipids in the blood of active-duty soldiers, they identified elevated Triglycerides (TG) and Diglycerides (DG) in subjects with mTBI + PTSD. Whereas, *APOE* ε4 carriers with mTBI showed elevated DGs (Huguenard et al.). This study suggests that examining peripheral TGs and DGs may be a useful biomarker for mTBI and PTSD, but ε4 status should be considered.

Changes in lipid composition may also be a useful predictor of Alzheimer's Disease progression (AD). Fonteh, Cipolla, et al., measured polyunsaturated fatty acids (PUFAs) in the cerebrospinal fluid (CSF) of participants that were either cognitively healthy with normal Aβ_42_/T-tau (established biomarkers of AD), cognitively healthy with pathological Aβ_42_/T-tau, or were diagnosed with AD. When normalized for the number of particles in the CSF, higher PUFA levels were observed in cognitively healthy individuals compared to subjects with AD, suggesting that higher PUFA levels may enhance cognitive resilience in the pre-symptomatic AD population ( Fonteh, Cipolla, et al.). These findings warrant further studies that examine whether PUFA composition can predict the transition from pre-symptomatic AD to AD and whether PUFA supplementation may prevent AD progression.

In a follow-up study, Fonteh, Chiang, et al., revealed further differences in the lipid composition of the CSF from cognitively healthy individuals, with either normal or pathological levels of Aβ_42_ and Tau or subjects with AD dementia. Cognitively healthy subjects with abnormal Aβ_42/_Tau showed elevated phosphatidylcholine glycerophospholipids (GPs) and higher sphingomyelin levels than those with AD, suggesting higher lipid levels in pre-symptomatic AD and lipolysis with AD progression (Fonteh, Chiang, et al.). In addition, the authors show that Phospholipase A_2_ (PLA_2_), which hydrolyzes membrane lipids, is increased in patients with dementia (Fonteh, Chiang, et al.). More work is needed to determine whether this is causative to the AD lipidome and whether altering PLA_2_ activity is a plausible strategy for treating AD.

AD treatment is particularly challenging since multiple biological, genetic, and lifestyle factors contribute to its neuropathogenesis. This issue is reviewed by Chew et al., in this Research Topic, where the authors highlight that altered lipid transport and metabolism is a common denominator driving the mechanisms leading to increased AD risk, such as blood-brain barrier function, amyloid precursor protein (APP) processing, myelination, membrane remodeling, receptor signaling, inflammation, oxidation, cardiovascular disease and energy balance. This comprehensive assessment of the current literature galvanizes the importance of interrogating the interaction between lipid metabolism and each facet of AD to help develop potential therapies that target lipid pathways (Chew et al.).

While changes in lipid composition may help diagnose and even treat NDs, the cellular processing of these lipids remains elusive, hindering the development of lipids as therapeutics. In recent years, microglia, the key immune effector cells of the CNS, have been critical to developing NDs. In a timely review, Loving and Bruce evaluate emerging evidence supporting the role of microglial subpopulations, defined by enhanced expression of lipoprotein processing machinery and increased phagocytosis, as potentially protective during development, damage, and disease. This review highlights the need for further studies that determine whether brain-derived lipoproteins and microglial lipoprotein receptors may be novel targets for treating NDs, such as AD and beyond.

Emerging evidence suggests that the cells of the CNS, including microglia, accumulate LDs during disease and aging. While LD biology has been well-characterized in peripheral metabolic tissues, the biology and significance of LDs within neurons and glia are relatively understudied. A review article in this Research Topic critically evaluates new research showing that LDs have diverse roles in the CNS, such as cellular fuel stores, markers of inflammation, signaling hubs, waste reservoirs, products of lysosomal degradation, and as hallmarks of aging (Farmer et al.). In light of these diverse roles, it is not surprising that LDs have been attributed to neurodegeneration and aberrant cerebral metabolism (Farmer et al.). However, further work is needed using improved imaging techniques (e.g., matrix-assisted laser desorption/ionization imaging mass spectrometry [MALDI-IMS]) to resolve region and cell-specific LD composition to further understand the contribution of LDs to NDs (Farmer et al.).

Many studies, including those outlined in this topic, support the hypothesis that supplementation with specific lipids (e.g., PUFAs) may improve cognitive function during aging and prevent the development of ND. However, we do not yet know how lipids are transferred from the blood to the brain. Thus, inconsistent outcomes following PUFA supplementation in AD patients likely relate to our incomplete understanding of lipid transport. In this current topic, Pifferi et al. carefully review our current understanding of lipid transport into and out of the CNS, paying particular attention to the differences between the blood-brain barrier (BBB) and the blood-cerebrospinal fluid (CSF) barrier (BCSFB). In addition, the authors highlight the understudied role of the choroid plexus (CP) within the BCSFB, a secretory tissue within the brain ventricles, as potentially critical to the maintenance of lipid and cholesterol transport (Pifferi et al.). Given the importance of CSF turnover and composition to the development of AD, an in-depth study of lipid transport mechanisms within the BCSFB is much needed (Pifferi et al.).

One transport mechanism that is critical for AD development yet also remains critically understudied is high-density lipoprotein (HDL) transport into the brain. Here, Van Valkeburgh et al. critically evaluate recent findings regarding the transport mechanisms of HDL particles (HDL-P), which are synthesized in both the brain and the periphery. HDL-associated apolipoproteins are some of the most important determinants of Alzheimer's disease (AD) pathology and vascular dementia. However, the extent to which HLD-P can exchange their protein and lipid components between the central nervous system and the systemic circulation remains unclear. Here, the authors delineate the structure and functions of HDL's apolipoproteins that influence brain amyloid metabolism and atherosclerosis; and explore how HDL can be modified to enhance its brain delivery with potential for treating brain neurodegenerative and vascular diseases (Van Valkenburgh et al.). Small HDL-P appear to possess properties that can favor their brain delivery, but further research is needed to understand their transport mechanisms (Van Valkenburgh et al.).

In conclusion, the Research Topic “*Lipid Metabolism and Transport in CNS Health and Disease*” is a collection of manuscripts highlighting the importance of lipid processing in brain structure and function. These manuscripts underscore what is known, what needs to be studied, and the benefits of lipid analyses in CNS diseases; to understand disease mechanisms, identify biomarkers, and discover novel treatments.

## Author Contributions

KB, HY, and AF wrote and edited the manuscript. All authors contributed to the article and approved the submitted version.

## Conflict of Interest

The authors declare that the research was conducted in the absence of any commercial or financial relationships that could be construed as a potential conflict of interest.

## Publisher's Note

All claims expressed in this article are solely those of the authors and do not necessarily represent those of their affiliated organizations, or those of the publisher, the editors and the reviewers. Any product that may be evaluated in this article, or claim that may be made by its manufacturer, is not guaranteed or endorsed by the publisher.

